# Impact of social problem-solving training on critical thinking and decision making of nursing students

**DOI:** 10.1186/s12912-020-00487-x

**Published:** 2020-10-07

**Authors:** Soleiman Ahmady, Sara Shahbazi

**Affiliations:** 1 Department of Medical Education, Virtual School of Medical Education and Management, Shahid Beheshti University of Medical Sciences, Tehran, Iran; 2 Virtual School of Medical Education and management, Shahid Beheshty University of Medical Sciences, Tehran, Iran; 3 Community-Oriented Nursing Midwifery Research Center, Shahrekord University of Medical Sciences, Shahrekord, Iran

**Keywords:** Social problem solving, Decision making, Critical thinking, Nursing, Student

## Abstract

**Background:**

The complex health system and challenging patient care environment require experienced nurses, especially those with high cognitive skills such as problem-solving, decision- making and critical thinking. Therefore, this study investigated the impact of social problem-solving training on nursing students’ critical thinking and decision-making.

**Methods:**

This study was quasi-experimental research and pre-test and post-test design and performed on 40 undergraduate/four-year students of nursing in Borujen Nursing School/Iran that was randomly divided into 2 groups; experimental (*n* = 20) and control (n = 20). Then, a social problem-solving course was held for the experimental group. A demographic questionnaire, social problem-solving inventory-revised, California critical thinking test, and decision-making questionnaire was used to collect the information. The reliability and validity of all of them were confirmed. Data analysis was performed using SPSS software and independent sampled T-test, paired T-test, square chi, and Pearson correlation coefficient.

**Results:**

The finding indicated that the social problem-solving course positively affected the student’ social problem-solving and decision-making and critical thinking skills after the instructional course in the experimental group (*P* < 0.05), but this result was not observed in the control group (*P* > 0.05).

**Conclusions:**

The results showed that structured social problem-solving training could improve cognitive problem-solving, critical thinking, and decision-making skills. Considering this result, nursing education should be presented using new strategies and creative and different ways from traditional education methods. Cognitive skills training should be integrated in the nursing curriculum. Therefore, training cognitive skills such as problem- solving to nursing students is recommended.

## Background

Continuous monitoring and providing high-quality care to patients is one of the main tasks of nurses. Nurses’ roles are diverse and include care, educational, supportive, and interventional roles when dealing with patients’ clinical problems [1, 2].

Providing professional nursing services requires the cognitive skills such as problem-solving, decision-making and critical thinking, and information synthesis [3].

Problem-solving is an essential skill in nursing. Improving this skill is very important for nurses because it is an intellectual process which requires the reflection and creative thinking [4].

Problem-solving skill means acquiring knowledge to reach a solution, and a person’s ability to use this knowledge to find a solution requires critical thinking. The promotion of these skills is considered a necessary condition for nurses’ performance in the nursing profession [5, 6].

Managing the complexities and challenges of health systems requires competent nurses with high levels of critical thinking skills. A nurse’s critical thinking skills can affect patient safety because it enables nurses to correctly diagnose the patient’s initial problem and take the right action for the right reason [4, 7, 8].

Problem-solving and decision-making are complex and difficult processes for nurses, because they have to care for multiple patients with different problems in complex and unpredictable treatment environments [9, 10].

Clinical decision making is an important element of professional nursing care; nurses’ ability to form effective clinical decisions is the most significant issue affecting the care standard. Nurses build 2 kinds of choices associated with the practice: patient care decisions that affect direct patient care and occupational decisions that affect the work context or teams [11–16].

The utilization of nursing process guarantees the provision of professional and effective care. The nursing process provides nurses with the chance to learn problem-solving skills through teamwork, health management, and patient care. Problem-solving is at the heart of nursing process which is why this skill underlies all nursing practices. Therefore, proper training of this skill in an undergraduate nursing program is essential [17].

Nursing students face unique problems which are specific to the clinical and therapeutic environment, causing a lot of stresses during clinical education. This stress can affect their problem- solving skills [18–21]. They need to promote their problem-solving and critical thinking skills to meet the complex needs of current healthcare settings and should be able to respond to changing circumstances and apply knowledge and skills in different clinical situations [22]. Institutions should provide this important opportunity for them.

Despite, the results of studies in nursing students show the weakness of their problem-solving skills, while in complex health environments and exposure to emerging diseases, nurses need to diagnose problems and solve them rapidly accurately. The teaching of these skills should begin in college and continue in health care environments [5, 23, 24].

It should not be forgotten that in addition to the problems caused by the patients’ disease, a large proportion of the problems facing nurses are related to the procedures of the natural life of their patients and their families, the majority of nurses with the rest of health team and the various roles defined for nurses [25].

Therefore, in addition to above- mentioned issues, other ability is required to deal with common problems in the working environment for nurses, the skill is “social problem solving”, because the term social problem-solving includes a method of problem-solving in the “natural context” or the “real world” [26, 27]. In reviewing the existing research literature on the competencies and skills required by nursing students, what attracts a lot of attention is the weakness of basic skills and the lack of formal and systematic training of these skills in the nursing curriculum, it indicates a gap in this area [5, 24, 25]. In this regard, the researchers tried to reduce this significant gap by holding a formal problem-solving skills training course, emphasizing the common social issues in the real world of work. Therefore, this study was conducted to investigate the impact of social problem-solving skills training on nursing students’ critical thinking and decision-making.

## Methods

### Setting and sample

This quasi-experimental study with pretest and post-test design was performed on 40 undergraduate/four-year nursing students in Borujen nursing school in Shahrekord University of Medical Sciences. The periods of data collection were 4 months.

According to the fact that senior students of nursing have passed clinical training and internship programs, they have more familiarity with wards and treatment areas, patients and issues in treatment areas and also they have faced the problems which the nurses have with other health team personnel and patients and their families, they have been chosen for this study. Therefore, this study’s sampling method was based on the purpose, and the sample size was equal to the total population. The whole of four-year nursing students participated in this study and the sample size was 40 members. Participants was randomly divided in 2 groups; experimental (*n* = 20) and control (n = 20).

The inclusion criteria to take part in the present research were students’ willingness to take part, studying in the four-year nursing, not having the record of psychological sickness or using the related drugs (all based on their self-utterance).

### Intervention

At the beginning of study, all students completed the demographic information’ questionnaire. The study’s intervening variables were controlled between the two groups [such as age, marital status, work experience, training courses, psychological illness, psychiatric medication use and improving cognitive skills courses (critical thinking, problem- solving, and decision making in the last 6 months)]. Both groups were homogeneous in terms of demographic variables (*P* > 0.05). Decision making and critical thinking skills and social problem solving of participants in 2 groups was evaluated before and 1 month after the intervention.

All questionnaires were anonymous and had an identification code which carefully distributed by the researcher.

To control the transfer of information among the students of two groups, the classification list of students for internships, provided by the head of nursing department at the beginning of semester, was used.

Furthermore, the groups with the odd number of experimental group and the groups with the even number formed the control group and thus were less in contact with each other.

The importance of not transferring information among groups was fully described to the experimental group. They were asked not to provide any information about the course to the students of the control group.

Then, training a course of social problem-solving skills for the experimental group, given in a separate course and the period from the nursing curriculum and was held in 8 sessions during 2 months, using small group discussion, brainstorming, case-based discussion, and reaching the solution in small 4 member groups, taking results of the social problem-solving model as mentioned by D-zurilla and gold fried [26]. The instructor was an assistant professor of university and had a history of teaching problem-solving courses. This model’ stages are explained in Table 1.

**Table 1:** Stages of D-zurilla and Gold fried social problem solving model

Stages	Title of stages	Actions
1	General orientation	• The ability to identify problem • Acknowledging the problem as a changeable potentially natural phenomenon • Believed to be effective in dealing with the problem solving framework • High self-efficiency expectations to implement stages of model • Accustomed to stop, think, and then making effort to solve a problem.
2	Defining and formulating the problem	Collection of all information available • Separation of facts is of the assumptions which require investigation • Analysis of the problem • Specifying the actual objectives.
3	Production of alternative solutions	To determine wide range of possible solutions • Ability to choose the most effective response to replies.
4	Decision making	• Predict probable consequences of each action • Paying due attention to the usefulness of these consequences.
5	Implementation of solution	• Execute the selected method
6	Review	• Observing the results of execution • Evaluation

All training sessions were performed due to the model, and one step of the model was implemented in each session. In each session, the teacher stated the educational objectives and asked the students to share their experiences in dealing to various workplace problems, home and community due to the topic of session. Besides, in each session, a case-based scenario was presented and thoroughly analyzed, and students discussed it.

### Instruments

In this study, the data were collected using demographic variables questionnaire and social problem- solving inventory – revised (SPSI-R) developed by D’zurilla and Nezu (2002) [26], California critical thinking skills test- form B (CCTST; 1994) [27, 28] and decision-making questionnaire.

SPSI-R is a self - reporting tool with 52 questions ranging from a Likert scale (1: Absolutely not – 5: very much).

The minimum score maybe 25 and at a maximum of 125, therefore:

- The score 25 and 50: weak social problem-solving skills.
- The score 50–75: moderate social problem-solving skills.
- The score higher of 75: strong social problem-solving skills.

The reliability assessed by repeated tests is between 0.68 and 0.91, and its alpha coefficient between 0.69 and 0.95 was reported [26]. The structural validity of questionnaire has also been confirmed. All validity analyses have confirmed SPSI as a social problem - solving scale.

In Iran, the alpha coefficient of 0.85 is measured for five factors, and the retest reliability coefficient was obtained 0.88. All of the narratives analyzes confirmed SPSI as a social problem- solving scale [29].

California critical thinking skills test- form B(CCTST; 1994): This test is a standard tool for assessing the basic skills of critical thinking at the high school and higher education levels (Facione & Facione, 1992, 1998) [27].

This tool has 34 multiple-choice questions which assessed analysis, inference, and argument evaluation. Facione and Facione (1993) reported that a KR-20 range of 0.65 to 0.75 for this tool is acceptable [27].

In Iran, the KR-20 for the total scale was 0.62. This coefficient is acceptable for questionnaires that measure the level of thinking ability of individuals.

After changing the English names of this questionnaire to Persian, its content validity was approved by the Board of Experts.

The subscale analysis of Persian version of CCTST showed a positive high level of correlation between total test score and the components (analysis, r = 0.61; evaluation, r = 0.71; inference, r = 0.88; inductive reasoning, r = 0.73; and deductive reasoning, r = 0.74) [28].

A decision-making questionnaire with 20 questions was used to measure decision-making skills. This questionnaire was made by a researcher and was prepared under the supervision of a professor with psychometric expertise. Five professors confirmed the face and content validity of this questionnaire. The reliability was obtained at 0.87 which confirmed for 30 students using the test-retest method at a time interval of 2 weeks. Each question had four levels and a score from 0.25 to 1. The minimum score of this questionnaire was 5, and the maximum score was 20 [30].

### Statistical analysis

For analyzing the applied data, the SPSS Version 16, and descriptive statistics tests, independent sample T-test, paired T-test, Pearson correlation coefficient, and square chi were used. The significant level was taken *P* < 0.05.

## Results

The average age of students was 21.7 ± 1.34, and the academic average total score was 16.32 ± 2.83. Other demographic characteristics are presented in Table 2.

**Table 2:** Demographic characteristics of participants in study

variable		Frequency and percentage
Gender	Female	24(67.5%)
	Male	16(32.5%)
Marital status	Married	3(7.5%)
	Single	37(92.5%)
Place of living	Home	5(12.5%)
	student dormitory	35(87.5%)
Place of living before university	Village/Town	23(57.5%)
	City	17(42.5%)

None of the students had a history of psychiatric illness or psychiatric drug use. Findings obtained from the chi-square test showed that there is not any significant difference between the two groups statistically in terms of demographic variables.

The mean scores in social decision making, critical thinking, and decision-making in whole samples before intervention showed no significant difference between the two groups statistically (*P* > 0.05), but showed a significant difference after the intervention (*P* < 0.05) (Table 3).

**Table 3:** Comparing mean change of social problem solving, critical thinking and decision making skill score before and after of intervention between experimental and control groups

Variable	Time of intervention	Mean ± sd		*p*-value
		Group		
		experimental	control	
Social problem solving	Before	63.28 ± 3.08	64.57 ± 2.71	0.651
	After	109.12 ± 2.64	65.36 ± 2.03	0.000
Critical thinking	Before	9.98 ± 2.24	10.25 ± 1.01	0.924
	After	14.36 ± 0.98	10.72 ± 0.78	0.000
Decision making	Before	13.72 ± 2.19	13.63 ± 1.92	0.850
	After	18.35 ± 1.04	14.05 ± 1.83	0.000

Scores in Table 4 showed a significant positive difference before and after intervention in the “experimental” group (*P* < 0.05), but this difference was not seen in the control group (*P* > 0.05).

**Table 4:** Comparing mean scores of social problem solving, critical thinking and decision making skill before and after of intervention in experimental and control groups

Time of intervention Variable	experimental		control	
	Mean ± sd		Mean ± sd	
	Before	After	Before	After
Social problem solving	63.28 ± 3.08	109.12 ± 2.64	64.57 ± 2.71	65.36 ± 2.03
p-value	0.000		0.675	
Critical thinking	9.98 ± 2.24	14.36 ± 0.98	10.25 ± 1.01	10.72 ± 0.78
p-value	0.000		0.547	
Decision making	13.72 ± 2.19	18.35 ± 1.04	13.63 ± 1.92	14.05 ± 1.83
p-value	0.000		0.592	

Among the demographic variables, only a positive relationship was seen between marital status and decision-making skills (r = 0.72, *P* < 0.05).

Also, the scores of critical thinking skill’ subgroups and social problem solving’ subgroups are presented in Tables 5 and 6 which showed a significant positive difference before and after intervention in the “experimental” group (P < 0.05), but this difference was not seen in the control group (*P* > 0.05).

**Table 5:** Comparing mean scores of critical thinking skill subgroups before and after of intervention in experimental and control groups

Time of intervention Variable	experimental		control	
	Mean ± sd		Mean ± sd	
	Before	After	Before	After
analysis	2.41 ± 2.06	6.32 ± 1.15	2.40 ± 1.11	2.26 ± 1.21
p-value	0.000		0.512	
evaluation	3.166 ± 2.037	7.08 ± 0.88	3.650 ± 1.386	3.846 ± 1.908
p-value	0.000		0.608	
inference	3 ± 1.362	6.24 ± 1.32	3.1 ± 1.372	2.76 ± 1.739
p-value	0.000		0.593

**Table 6:** Comparing mean scores of social problem solving subgroups before and after of intervention in experimental and control groups

functioning	Time of intervention Variable	experimental		control	
		Mean ± sd		Mean ± sd	
		Before	After	Before	After
Adaptive problem solving	Positive problem orientation	11.52 ± 2.03	13.97 ± 1.68	10.12 ± 2.02	11.10 ± 2.13
	p-value	0.000		0.702	
	rational problem solving	24.29 ± 4.29	28.32 ± 1.99	22.67 ± 3.85	21.98 ± 2.00
	p-value	0.000		0.830	
maladaptive problem solving	negative problem orientation	14.7 ± 2.53	7.05 ± 2.12	13.48 ± 3.25	14.11 ± 2.87
	p-value	0.000		0.512	
	impulsivity-carelessness style	13.02 ± 2.83	10.26 ± 1.27	12.99 ± 2.95	13.00 ± 1.74
	p-value	0.000		0.608	
	avoidance style	12.72 ± 3.05	9.52 ± 2.04	13.07 ± 2.90	13.20 ± 2.53
	p-value	0.000		0.593	

## Discussion

In the present study conducted by some studies, problem-solving and critical thinking and decision-making scores of nursing students are moderate [5, 24, 31].

The results showed that problem-solving skills, critical thinking, and decision-making in nursing students were promoted through a social problem-solving training course. Unfortunately, no study has examined the effect of teaching social problem-solving skills on nursing students’ critical thinking and decision-making skills.

Altun (2018) believes that if the values of truth and human dignity are promoted in students, it will help them acquire problem-solving skills. Free discussion between students and faculty on value topics can lead to the development of students’ information processing in values. Developing self-awareness increases students’ impartiality and problem-solving ability [5]. The results of this study are consistent to the results of present study.

Erozkan (2017), in his study, reported there is a significant relationship between social problem solving and social self-efficacy and the sub-dimensions of social problem solving [32]. In the present study, social problem -solving skills training has improved problem -solving skills and its subdivisions.

The results of study by Moshirabadi (2015) showed that the mean score of total problem-solving skills was 89.52 ± 21.58 and this average was lower in fourth-year students than other students. He explained that education should improve students’ problem-solving skills. Because nursing students with advanced problem-solving skills are vital to today’s evolving society [22]. In the present study, the results showed students’ weakness in the skills in question, and holding a social problem-solving skills training course could increase the level of these skills.

Çinar (2010) reported midwives and nurses are expected to use problem-solving strategies and effective decision-making in their work, using rich basic knowledge.

These skills should be developed throughout one’s profession. The results of this study showed that academic education could increase problem-solving skills of nursing and midwifery students, and final year students have higher skill levels [23].

Bayani (2012) reported that the ability to solve social problems has a determining role in mental health. Problem-solving training can lead to a level upgrade of mental health and quality of life [33]; These results agree with the results obtained in our study.

Conducted by this study, Kocoglu (2016) reported nurses’ understanding of their problem-solving skills is moderate. Receiving advice and support from qualified nursing managers and educators can enhance this skill and positively impact their behavior [31].

Kashaninia (2015), in her study, reported teaching critical thinking skills can promote critical thinking and the application of rational decision-making styles by nurses.

One of the main components of sound performance in nursing is nurses’ ability to process information and make good decisions; these abilities themselves require critical thinking. Therefore, universities should envisage educational and supportive programs emphasizing critical thinking to cultivate their students’ professional competencies, decision-making, problem-solving, and self-efficacy [34].

The study results of Kirmizi (2015) also showed a moderate positive relationship between critical thinking and problem-solving skills [35].

Hong (2015) reported that using continuing PBL training promotes reflection and critical thinking in clinical nurses. Applying brainstorming in PBL increases the motivation to participate collaboratively and encourages teamwork. Learners become familiar with different perspectives on patients’ problems and gain a more comprehensive understanding. Achieving these competencies is the basis of clinical decision-making in nursing. The dynamic and ongoing involvement of clinical staff can bridge the gap between theory and practice [36].

Ancel (2016) emphasizes that structured and managed problem-solving training can increase students’ confidence in applying problem-solving skills and help them achieve self-confidence. He reported that nursing students want to be taught in more innovative ways than traditional teaching methods which cognitive skills training should be included in their curriculum. To this end, university faculties and lecturers should believe in the importance of strategies used in teaching and the richness of educational content offered to students [17].

The results of these recent studies are adjusted with the finding of recent research and emphasize the importance of structured teaching cognitive skills to nurses and nursing students.

Based on the results of this study on improving critical thinking and decision-making skills in the intervention group, researchers guess the reasons to achieve the results of study in the following cases:

In nursing internationally, problem-solving skills (PS) have been introduced as a key strategy for better patient care [17]. Problem-solving can be defined as a self-oriented cognitive-behavioral process used to identify or discover effective solutions to a special problem in everyday life. In particular, the application of this cognitive-behavioral methodology identifies a wide range of possible effective solutions to a particular problem and enhancement the likelihood of selecting the most effective solution from among the various options [27].

In social problem-solving theory, there is a difference among the concepts of problem-solving and solution implementation, because the concepts of these two processes are different, and in practice, they require different skills.

In the problem-solving process, we seek to find solutions to specific problems, while in the implementation of solution, the process of implementing those solutions in the real problematic situation is considered [25, 26].

The use of D’zurilla and Goldfride’s social problem-solving model was effective in achieving the study results because of its theoretical foundations and the usage of the principles of cognitive reinforcement skills. Social problem solving is considered an intellectual, logical, effort-based, and deliberate activity [26, 32]; therefore, using this model can also affect other skills that need recognition.

In this study, problem-solving training from case studies and group discussion methods, brainstorming, and activity in small groups, was used.

There are significant educational achievements in using small- group learning strategies. The limited number of learners in each group increases the interaction between learners, instructors, and content. In this way, the teacher will be able to predict activities and apply techniques that will lead students to achieve high cognitive taxonomy levels. That is, confront students with assignments and activities that force them to use cognitive processes such as analysis, reasoning, evaluation, and criticism.

In small groups, students are given the opportunity to the enquiry, discuss differences of opinion, and come up with solutions. This method creates a comprehensive understanding of the subject for the student [36].

According to the results, social problem solving increases the nurses’ decision-making ability and critical thinking regarding identifying the patient’s needs and choosing the best nursing procedures. According to what was discussed, the implementation of this intervention in larger groups and in different levels of education by teaching other cognitive skills and examining their impact on other cognitive skills of nursing students, in the future, is recommended.

Social problem- solving training by affecting critical thinking skills and decision-making of nursing students increases patient safety. It improves the quality of care because patients’ needs are better identified and analyzed, and the best solutions are adopted to solve the problem.

In the end, the implementation of this intervention in larger groups in different levels of education by teaching other cognitive skills and examining their impact on other cognitive skills of nursing students in the future is recommended.

### Study limitations

This study was performed on fourth-year nursing students, but the students of other levels should be studied during a cohort from the beginning to the end of course to monitor the cognitive skills improvement.

## Conclusion

The promotion of high-level cognitive skills is one of the main goals of higher education. It is very necessary to adopt appropriate approaches to improve the level of thinking. According to this study results, the teachers and planners are expected to use effective approaches and models such as D’zurilla and Goldfride social problem solving to improve problem-solving, critical thinking, and decision-making skills. What has been confirmed in this study is that the routine training in the control group should, as it should, has not been able to improve the students’ critical thinking skills, and the traditional educational system needs to be transformed and reviewed to achieve this goal.

## Data Availability

The datasets used and analyzed during the present study are available from the corresponding author on reasonable request.

## Abbreviations

CCTST: California critical thinking skills test
SPSI-R: Social problem-solving inventory – revised
